# Direct puncture embolisation of the non-coil-embolised internal iliac artery post EVAR - a novel use of the Angio-Seal closure device

**DOI:** 10.1186/s42155-018-0012-6

**Published:** 2018-06-28

**Authors:** Prashant Ravindran Menon, Sanjay Agarwal, Owen Rees

**Affiliations:** 1 North Wales School of Radiology, Department of Radiology, Wrexham Maelor Hospital, Croesnewydd Road, Wrexham, LL13 7TD UK; 2 Department of Radiology, Wrexham Maelor Hospital, Croesnewydd Road, Wrexham, LL13 7TD UK; 3 Department of Radiology, Glan Clwyd Hospital, Rhuddlan Road, Bodelwyddan, Rhyl LL18 5UJ UK

**Keywords:** Angioseal, Aortic interventions, Type II endoleak, Internal iliac artery coiling pre EVAR

## Abstract

**Background:**

Coil embolisation of the internal iliac arteries prior to EVAR is considered standard treatment to prevent a type 2 endoleak when extending an iliac limb into the EIA. Type 2 endoleaks that arise from a non-coil-embolised internal iliac artery can be challenging to treat due to difficult access.

**Case presentation:**

We present a case of a type 2 endoleak from the internal iliac artery that was not coiled prior to EVAR. This was treated with retrograde embolisation of the internal iliac artery via direct puncture of a branch from the buttock and closure of the arteriotomy was achieved using an Angio-Seal (Terumo) device that was deployed in an off-label manner to allow visualisation.

**Conclusion:**

This is a viable technique for treating type 2 endoleaks when antegrade access to the internal iliac artery is lost due to the presence of the stent graft and the arteriotomy can be safely closed with an Angio-Seal.

## Background

Coil embolisation of the internal iliac artery as an adjunct to endovascular stent grafting is common practice for treating abdominal aortic aneurysms in patients requiring extension of a stent graft limb into the external iliac artery (EIA) (Chun et al. 2014). This may be due to an aneurysmal or abnormally short common iliac artery (CIA). The reasoning behind the procedure is to prevent Type 2 endoleaks arising from the internal iliac artery. Embolisation is normally performed prior to the endovascular aneurysm repair (EVAR) while there is antegrade access to the IIA.

We present a case of a type 2 endoleak from an IIA that was not coiled prior to EVAR and treatment of this with retrograde embolization of the IIA via direct puncture of a branch via the buttock. Closure of the arteriotomy was with an Angio-Seal (Terumo) closure device, deployed in an off-label manner.

## Case presentation

**Fig. 1:**
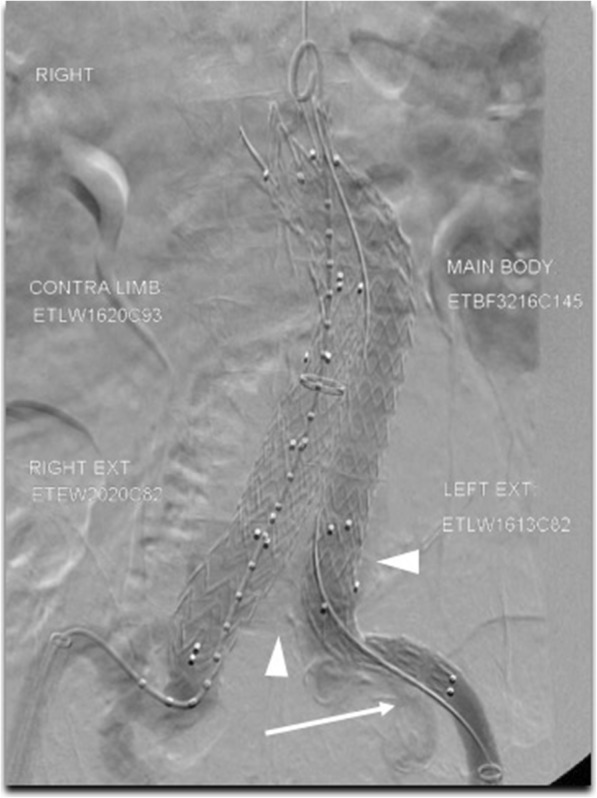
Static image from angiogram post stent graft deployment. Arrowheads demonstrate type II endoleak within the aneurysm sac. Arrow demonstrates patent left IIA

An 80-year-old Caucasian man who initially presented with back pain was found to have a 9.8 cm infrarenal abdominal aortic aneurysm on computed tomography (CT). This was suitable for endovascular aneurysm repair (EVAR) but the left common iliac artery (CIA) was short, necessitating the extension of the left iliac limb into the external iliac artery (EIA). The decision was made to proceed with an on table coiling of the left internal iliac artery (IIA) and EVAR. However, a tortuous right CIA made coiling of the left IIA extremely difficult and following a prolonged attempt, the decision was made to proceed with EVAR without coiling the IIA (Fig. 1).

On a 1-month follow up CT the left limb extension had disengaged from the main body, resulting in a large Type 3 endoleak. The disengagement was presumed to be due to the tortuosity of a heavily calcified external iliac artery returning to its anatomical position on removal of the Meir wire (Boston Scientific). The left IIA remained patent and acted as an outflow for the type 3 endoleak. The left limb was then realigned successfully with termination of the type 3 endoleak.

**Fig. 2:**
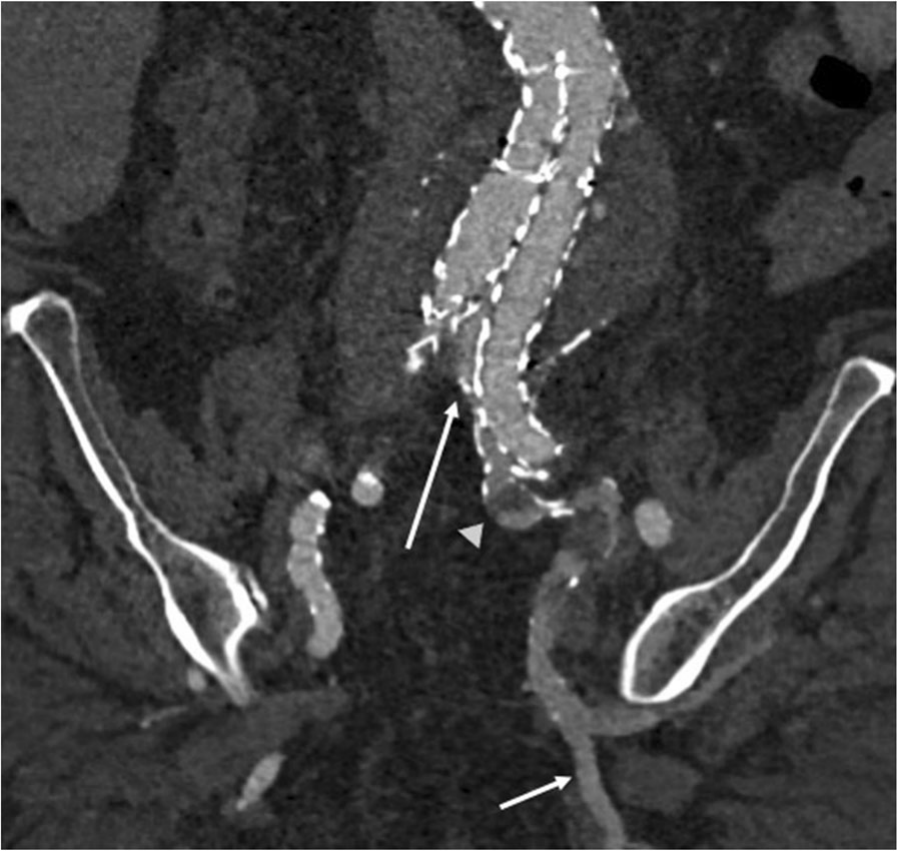
Reformatted CT angiogram image showing contrast filling of left internal iliac artery (arrowhead) and type II endoleak in the aneurysm sac (short arrow). The anterior division of the left IIA is demonstrated with the long arrow

On a follow up CT 1 month post repair, the left IIA remained patent with a large type 2 endoleak demonstrated but the sac size remained static (Fig. 2). The failure of the IIA to occlude was presumed due to the cavity created within the aneurysm sac from the type 3 endoleak. Due to the large size of the aneurysm and the relatively large size of the type 2 endoleak the decision was made at multi-disciplinary team meeting to embolise the type 2 endoleak.

**Fig. 3:**
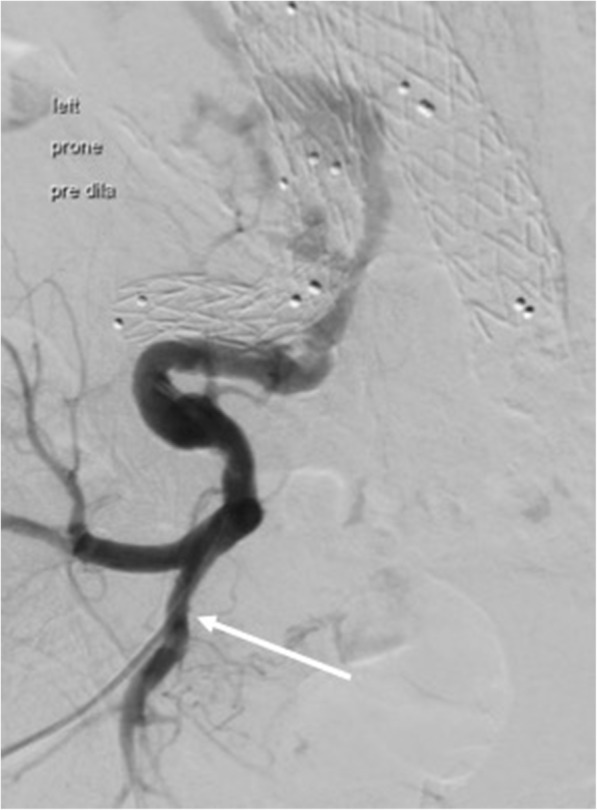
Angiogram via a 4 Fr catheter inserted in the anterior branch of the left IIA (arrow) demonstrating type II endoleak within the aneurysm sac (arrowhead)

**Fig. 4:**
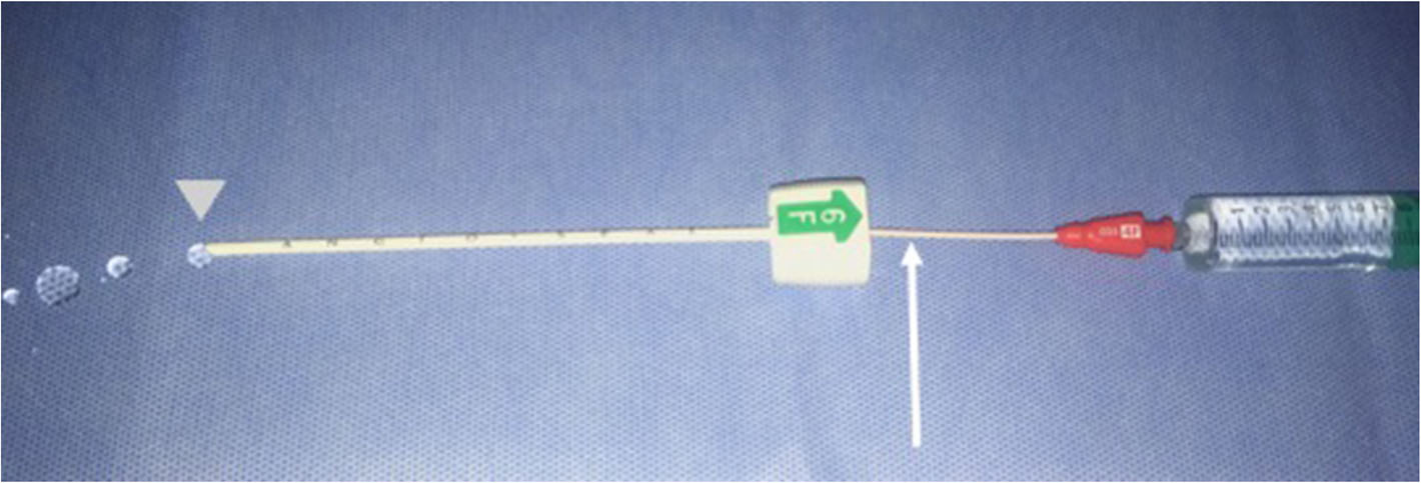
Photograph showing 4F dilator inserted into the angioseal sheath (arrow) to illustrate the method of injecting contrast through the sheath to visualise the tip on fluoroscopy (arrowhead)

**Fig. 5:**
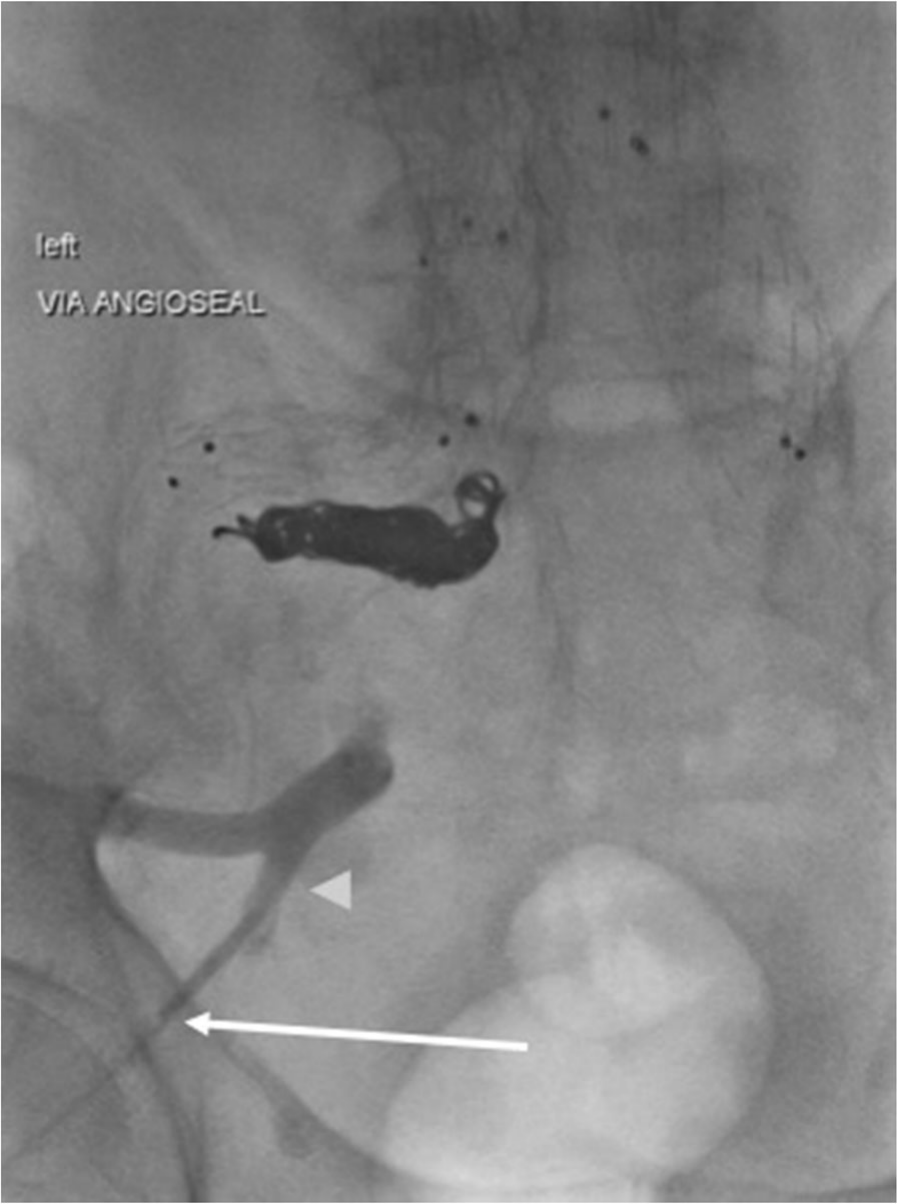
Angiogram via 4Fr dilator inserted through AngioSeal sheath (arrow) to visualise angioseal sheath tip (arrowhead)

As antegrade access to the left IIA was not possible due to the presence of the stent graft, an attempt was made to access the internal iliac artery via the buttock. Direct sac puncture was not possible as the endoleak was located within the pelvis and surrounded by bony structures and pelvic viscera. The intention was to puncture the posterior division of the IIA but visualisation with ultrasound was limited and a vessel was punctured, which on subsequent angiography was shown to be the anterior division of the left IIA (Fig. 3). The left IIA was embolised using Spirali (Pyramed) coils in a retrograde manner. Due to the absence of a solid structure which to compress the access vessel against, especially as the anterior division of the IIA had been punctured, the arteriotomy needed to be closed using an Angio-Seal (Terumo). Correct intraluminal placement of the Angio-Seal (Terumo) is dependent on backflow of blood into the Angio-Seal (Terumo) sheath from the artery. However, as the left IIA had been embolised, backflow from the Angio-Seal (Terumo) was non-existent. The angioseal was not visualised on ultrasound and therefore a 4 French dilator was inserted within the Angio-Seal (Terumo) sheath through which contrast was injected to directly visualise the tip to the sheath within the artery (Fig. 4). The sheath was then withdrawn until just inside the vessel lumen and the plug deployed with immediate haemostasis (Fig. 5). This was an off-label use of the Angio-Seal (Terumo).

A follow up CT 1 month post procedure revealed resolution of the Type 2 endoleak.

## Discussion

The need for IIA coiling prior to EVAR is not quite clear in the literature although this remains common practice along with the use of iliac branched devices. Wyers et al. 2002 have suggested that covering the IIA origin with the stent graft will result in occlusion of the IIA in most cases without the need for coiling, but only when there is adequate oversizing of the stent graft.

Retrograde access to the IIA is rarely required, with only a few cases reported in the literature (Aberhalden et al. 2012), (Kabutey et al. 2014). Werner-Gibbings et al. 2013 used a superior gluteal approach to treat a type 2 endoleak following EVAR. Previous approaches have used puncture of the gluteal branches to obtain access to the IIA. Kabutey et al. 2014 have used the gluteal artery approach to coil bilateral IIA aneurysms. In that case, haemostasis was achieved with manual compression. To our knowledge, this is the first published case to use an Angio-Seal (Terumo) to close the arteriotomy following retrograde embolization of the IIA. The intention in this case was to puncture a posterior division branch of the IIA to allow manual compression at the end of the procedure, however visualisation with ultrasound was extremely limited and this resulted in puncture of the anterior division. The decision was made to proceed with embolisation and use a closure device to aid in haemostasis due to the lack of a bony structure for compression. The technique described herein, using a 4 French dilator within the sheath to visualise the artery is safe and effective in any scenario where there is no backflow of blood from the vessel. This is an off-label use of the Angio-Seal (Terumo) device.

## Conclusion

We have shown that in the treatment of Type 2 endoleaks from a non-coil-embolised IIA, retrograde access via percutaneous puncture through the buttock is a viable option and that the arteriotomy can be safely closed using an Angio-Seal (Terumo) using the off-label technique described.
